# Mechanisms for the inhibition of amyloid aggregation by small ligands

**DOI:** 10.1042/BSR20160101

**Published:** 2016-09-29

**Authors:** Matteo Ramazzotti, Fabrizio Melani, Laura Marchi, Nadia Mulinacci, Stefano Gestri, Bruno Tiribilli, Donatella Degl'Innocenti

**Affiliations:** * Dipartimento di Scienze Biomediche Sperimentali e Cliniche, Università degli Studi di Firenze, viale Morgangi 50, 50134 Firenze, Italy; † Dipartimento di Neuroscienze, Psicologia, Area del Farmaco e Salute del Bambino, Università degli Studi di Firenze, Viale Pieraccini 6, 50139 Firenze, Italy; ‡ Liceo Scientifico Statale N. Copernico, via Borgovalsugana 63, 59100 Prato, Italy; § ISC-CNR–Istituto dei Sistemi Complessi, Consiglio Nazionale delle Ricerche Sede di Firenze, Via Madonna del Piano 10, 50019 Sesto Fiorentino, Firenze, Italy

**Keywords:** HEWL, contact map, energy, MD, molecular modelling, polyphenols

## Abstract

The formation of amyloid aggregates is the hallmark of systemic and neurodegenerative disorders, also known as amyloidoses. Many proteins have been found to aggregate into amyloid-like fibrils and this process is recognized as a general tendency of polypeptides. Lysozyme, an antibacterial protein, is a well-studied model since it is associated in human with systemic amyloidosis and that is widely available from chicken eggs (HEWL, hen egg white lysozyme). In the present study we investigated the mechanism of interaction of aggregating HEWL with rosmarinic acid and resveratrol, that we verified to be effective and ineffective, respectively, in inhibiting aggregate formation. We used a multidisciplinary strategy to characterize such effects, combining biochemical and biophysical methods with molecular dynamics (MD) simulations on the HEWL peptide 49–64 to gain insights into the mechanisms and energy variations associated to amyloid formation and inhibition. MD revealed that neither resveratrol nor rosmarinic acid were able to compete with the initial formation of the β-sheet structure. We then tested the association of two β-sheets, representing the model of an amyloid core structure. MD showed that rosmarinic acid displayed an interaction energy and a contact map comparable to that of sheet pairings. On the contrary, resveratrol association energy was found to be much lower and its contact map largely different than that of sheet pairings. The overall characterization elucidated a possible mechanism explaining why, in this model, resveratrol is inactive in blocking fibril formation, whereas rosmarinic acid is instead a powerful inhibitor.

## Abstract (precis)

This work investigates by biochemical, biophysical and MD techniques the opposite anti-amyloid properties of resveratrol and rosmarinic acid on the aggregation of hen egg white lysozyme (HEWL). Differences in association energy and contact maps were found that explain the different behaviours.

## INTRODUCTION

Amyloid aggregation can be described as a degenerative process characterized by the deposition at tissue levels of organized insoluble super-molecular protein assemblies with a typical cross-β secondary structure. Such degeneration give rise to amyloidoses, a composite range of diseases classically divided into neurodegenerative (e.g. Alzheimer's disease, Parkinson's disease, etc.) and systemic (e.g. cystic fibrosis, light chain amyloidosis). More than 20 different human proteins, intact, mutated or fragmented, proved their amyloidogenicity *in vivo*, among which Aβ-peptide (in Alzheimer's disease), α-synuclein (in Parkinson's disease), islet amyloid polypeptide (in type 2 diabetes), β2-microglobulin (in dialysis-related amyloidosis), light chains of immunoglobulins or variants of human lysozyme [1,2]. It is nowadays widely accepted that amyloid aggregation is a general tendency of polypeptide chains [3–5] that, in fact, may be induced to form amyloid aggregation in appropriate conditions [6].

**Figure 1:**
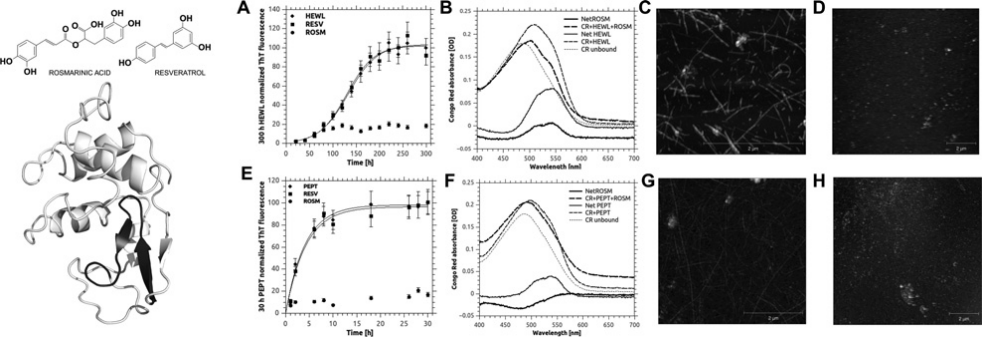
Rosmarinic acid inhibits amyloid-like aggregation of HEWL and HEWL 49–64 peptide Column 1: Structures of rosmarinic acid, resveratrol and HEWL (taken from PDB 2VB1). In the latter, the α-domain (top) and the β-domain (bottom) where the 49–64 peptide is located (highlighted in dark grey) are shown. Columns 2–5, top row: effect of rosmarinic acid and resveratrol on full-length HEWL. (**A**) Aggregation kinetic followed by ThT fluorescence at 485 nm. (**B**) CR assay at 300 h aggregation (ThT plateau). (**C**) AFM image (10 μm × 10 μm) of HEWL after 300 h aggregation. (**D**) AFM image (10 μm × 10 μm) of HEWL incubated with rosmarinic acid after 300 h aggregation. Columns 2–5, bottom row: effect of rosmarinic acid and resveratrol on 49–64 HEWL peptide. (**E**) Aggregation kinetic followed by ThT fluorescence at 485 nm. (**F**) CR assay at 30 h aggregation (ThT plateau). (G) AFM image (10 μm × 10 μm) of HEWL peptide after 30 h aggregation. (**H**) AFM image (10 μm × 10 μm) of HEWL peptide incubated with rosmarinic acid after 300 h aggregation. In (**A**) and (**E**) error bars represent S.D. of at least three independent experiments. Other panels show representative images of the corresponding experiments.

Lysozyme, a 130 residues long antibacterial enzyme widely distributed in different tissues, organs and external secretions, has been highlighted as an interesting model for the study of amyloid aggregation. Although wild-type lysozyme is not directly involved in amyloid diseases [7], several naturally occurring mutants (e.g. Ile56Thr, Phe57Ile, Trp64Arg and Asp67His) are connected with familial non-neuropathic systemic amyloidoses [8]. In addition, wild type lysozyme either from human, horse or chicken, under favourable conditions, is able to form amyloid fibrils *in vitro* [9–11]. In this work we used the hen egg white lysozyme (HEWL–14.3 kDa, 129 amino acids, 40% identity with the human form, fold α + β, see Figure 1), that has been shown to undergo amyloid-like aggregation through a heat treatment in acidic conditions [12]. It has been demonstrated that high temperatures and low pH induce the breakage of X-Asp peptide bonds, leading to the formation of peptide fragments with a high tendency to form amyloid aggregates [11] and amyloid-like fibrils within few days after the initial exposure. In addition, it has been demonstrated a direct toxic effect of HEWL aggregates added to cell cultures or injected in rat brains, mimicking the toxic effect of Aβ-peptide [13]. Previous studies on HEWL aggregation identified that, among the peptide segments involved in the formation of amyloid-like cores after fragmentation, the peptide 49–64 (originally located in the β-domain, see Figure 1) is particularly prone to aggregation even in mild conditions [11]. In fact, the aggregation kinetic of HEWL peptide 49–64 (hereinafter simply HEWL peptide) shows a single exponential shape, reaching a plateau in 15–20 h and lacks a lag phase, indicating that amyloid seeds are present since the earliest moment of aggregation. A shorter region within such peptide (54–62, called peptide K) has been widely investigated in order to identify the determinants of amyloidogenicity [14]. The Trp residue in position 62 was found to be fundamental, preceded by a stretch containing hydrophobic residues. More recently, this hydrophobic stretch was further identified as having sequence IFQINS in human lysozyme, and it was shown that it could spontaneously self-assemble into amyloid-like fibrils, whose structure was determined by crystallography [15]. All the reported studies demonstrate that HEWL is an established optimal model system for studying amyloid.

In this work we approached the topic of amyloid assembly and inhibition using molecular dynamics (MD). Our strategy was in part inspired by the recent work of Zhang et al. [16] that studied the interaction of β-sheets in IAPP by juxtaposing units and evaluating the energies and most favourable poses by MD. MD is widely used in drug design and, although admittedly limited due to the number of chemical approximations needed to operate calculations in a reasonable time [17], it offers the possibility of simulating the motion of molecules in force fields, that guide the energetic relationships among atoms and in turn evaluate the energy of a system or part of it. Importantly, MD treats every functional group (e.g. protein side chains or even backbones) or ligand as a dynamic element able to move in a constrained environment and therefore establish chemical and geometrical adaptations. From a chemical perspective, and based on recent trends, MD surpasses classical docking strategies and represents one of the most innovative tools in drug design [18].

A huge body of literature have documented that herbal extracts or common dietary elements such as wine [19] or green-tea [20] are of great benefit to general human health, mainly due to their antioxidant power [21,22]. Among the molecules proposed to be essential for achieving such effects there are polyphenols [23], a wide and heterogeneous group of substances well characterized in terms of structure [24] and beneficial properties. Besides protecting cells from oxidative stress, several phenolic compounds recently proved to be effective in inhibiting amyloid aggregation in various disease-related proteins such as α-synuclein, Aβ peptide, IAPP or transthyretin, with proposed specific action mechanisms independent on their antioxidant properties [25–28].

In this work we started from preliminary (unpublished data from the same authors) studies on HEWL amyloid inhibition by a battery of polyphenols representative of several different classes and we selected two well-known polyphenols, namely resveratrol (a typical stilbenoid of grapes and wine) and rosmarinic acid (the main aromatic compound of culinary herbs such as basil, sage and, more representatively, rosemary), as candidate molecules for the present study. In fact, using HEWL and HEWL 49–64 peptide (hereinafter simply HEWL peptide) as model systems of amyloid, we found that the latter was an effective anti-aggregation compound, as in other amyloid systems [29–32], whereas the former did not interfere at all with such process, contrarily to what observed in other systems, the most noticeable being the Aβ peptide [25,33,34]. In addition, these two polyphenols proved not to interfere with the Thioflavin-T (ThT) assay, that is not so frequent due to the limited solubility and the competitive effects with ThT of many polyphenolic compounds.

MD on a putative, energetically coherent reconstructed amyloid core of the HEWL peptide was eventually applied to highlight a possible mechanism leading to amyloid hampering by polyphenols and explain the experimental results.

## MATERIALS AND METHODS

### Materials

Reagents and chemicals, unless otherwise specified, were purchased from Sigma–Aldrich and used without further purification. Sigma–Aldrich also provided both the HEWL (code L6876) and the HEWL 49–64 peptide that was synthesized with purity >80% (ESI mass 1883±0.1 Da). This peptide had the same sequence as residues 49–64 of HEWL (GSTDYGILQINSRWWC), with the exception of last cysteine residue (C64 in HEWL) that was changed to serine to prevent disulfide bond formation as it occurs in the native protein [35]. Resveratrol and rosmarinic acid were from Extrasynthese.

### Aggregation conditions

To achieve a complete amyloid-like aggregation, full length HEWL or HEWL peptide at a concentration of 1 mM were incubated at 65°C in 10 mM HCl solutions (pH 2.0). Aggregation time (time to achieve a plateau, according to ThT measurements) was up to 10 days and 20–30 h, for full length HEWL or HEWL peptide respectively [35]. Polyphenols were dissolved in DMSO at 100 mM and added at 1:1 molar ratio to freshly dissolved HEWL or HEWL peptide. Control aggregation tests for both HEWL and HEWL peptide were also performed in presence of 1–5% v/v DMSO in order to exclude possible effects of DMSO on aggregation.

### Thioflavin-T assay

Aggregation kinetics was monitored by ThT assay [36]. For each measurement session, 0.25 mM ThT was freshly prepared from 25 mM stock solutions in 25 mM sodium phosphate buffer, pH 6.0. 495 μl of the 1:100 diluted ThT solution were added to a sample volume of 5 μl. ThT fluorescence was measured at 25°C in a 0.1 cm path quartz cuvette (Hellma) on a RF-5000 fluorimeter (Shimadzu) using 440 nm (5 nm slit) and 485 nm (5 nm slit) as excitation and emission wavelength respectively. A baseline measurement of fresh 100-fold diluted stock solution of ThT incubated with the HCl solutions alone or in presence of variable amounts of polyphenols at 10 μM concentration was used to correct sample signals.

ThT fluorescence measurements of aggregation kinetics on HEWL, both alone or in the presence of polyphenols and DMSO, were normalized to signals at 300 h and used to extrapolate a plateau signal thanks to a non-linear curve fitting using the eqn (1)

1 $$F=A_{i}+\left(A_{f}-A_{i}\right)/\left(l+\exp\left(\left(t_{1/2}-t\right)k_{\mathrm{agg}}\right)\right)$$

where *F* was the normalized fluorescence, *A*_i_ was the initial fluorescence, *A*_f_ the final fluorescence at plateau, *t* the incubation time, *t*_1/2_ the mid-point of aggregation and *k*_agg_ the apparent aggregation rate constant [37]. When polyphenols were added, the plateau values (*F*) were then used to calculate the percentage of inhibition with respect to HEWL control.

Similarly to HEWL, the HEWL peptide ThT fluorescence signals were normalized at 30 h and fitted to a mono-exponential curve using the equation (2)

2 $$F=A-A\;\exp(-k_{\mathrm{agg}}t)$$

where *F* was the normalized fluorescence, *A* was the total fluorescence, *t* the incubation time and *k*_agg_ the apparent aggregation rate constant.

### Congo red assay

For Congo red (CR) binding assays [38], a 50 mM CR solution was freshly prepared in 5 mM sodium phosphate buffer at pH 7.4 containing 150 mM NaCl and filtered through a 0.2-μm filter. Prior to use, CR solution was diluted 50-fold in the same buffer. Absorption spectra (400–700 nm) were recorder for (A) the buffer alone, (B) 440 μl buffer plus 60 μl aggregate solution, (C) 440 μl CR solution plus 60 μl HCl solution and (D) 440 μl CR solution plus 60 μl fibril solution. All these spectra were background-corrected by subtracting A and the difference spectra were calculated according to R=D − C − B. Samples were considered to contain amyloid material if the resulting difference spectra showed a peak at 540 nm, indicating a red shift typical of amyloid-bound CR. All spectra were recorded in a Pharmacia Biotech Ultrospec 2000 UV/vis spectrophotometer, using a low volume 0.5 cm path quartz cuvette (Hellma).

### CD

HEWL peptide aggregation, with or without polyphenols and DMSO, was also monitored with CD using a Jasco J-810 spectropolarimeter (Hachioji, Japan) equipped with a thermostatic water bath. CD spectra from 250 to 190 nm were recorded in HCl 10 mM at 25°C with 0.15 mg/ml protein concentration, in a 0.1 cm path length cuvette (Hellma).

### AFM

For the AFM analysis, HEWL or HEWL peptide samples at ThT plateau were agitated to ensure an effective sampling of the whole aggregated population, then they were diluted 1:100 or 1:200 in HCl 10 mM and a 5 μl drop was laid on to a freshly cleaved mica disc for approximately 2 min. Excess of sample was removed by washing twice with 1 ml of bi-distilled water, then the preparation was dried with a soft nitrogen flow. AFM experiments were performed in air, in non-contact mode, using a PicoSPM microscope equipped with AAC-Mode controller (Keysight Technologies, formerly Molecular Imaging). The probes were non-contact Silicon cantilevers (model NSG-01, NT-MDT Co., Moscow, Russia) with 150 kHz typical resonance frequency. Scanner calibration was periodically checked by means of a reference grid (TGZ02 by MikroMash) with known pitch 3 μm and step height 100 nm. Images were processed and analysed with Gwyddion v. 2.3.4 [39].

### Molecular modelling

The model amyloid core was tentatively created and refined with Discovery Studio Viewer Pro (DSV, Accelrys), AutoDock [40] and the Amber Molecular Dynamics suite v. 9.0 [41].

The HEWL peptide was modelled in DSV to achieve α-helix and β-strand conformations, whereas the native conformation was taken from the PDB structure 2VB1 [42] of the full length HEWL (basically a β-turn-β conformation). Assemblies were created by juxtaposing peptide monomers, previously described as being part of the amyloid core during HEWL aggregation and also used for the biophysical experiments detailed above.

### MD

The partial atomic charges for polyphenols were derived using the AM1-BCC method implemented in the ANTECHAMBER suite [43]. The energy minimizations and MD were carried out using the SANDER module of Amber 9 (University of California, San Francisco, USA) [41] with the GAFF [44] and ff99SB force fields. MD simulations were performed in implicit solvent using the generalized born surface area (GBSA) at constant pH. The constant pH MD method has been implemented in SANDER by Mongan et al. [45]. Before the dynamic simulation, 100 steps of steepest-descent and 900 steps of conjugate-gradient minimization on the entire complex were performed with a modified GB model (igb=2) [46], the surface area was computed and included in the solvation term, and a cutoff of 30 Å for non-bonded interactions was used. The system was then heated from 0 to 333 K in 5 ps by holding the complex fixed with a harmonic constraint of strength of 0.1 kcal/mol per Å^2^. In order to equilibrate the system, the complex was constrained with strength of 0.05 kcal/mol per Å^2^ and 50 ps dynamic simulations were performed at a constant temperature of 333 K with SHAKE turned on for bonds involving hydrogens, allowing a time-step of 2.0 fs. After this step, 1 ns of dynamic simulation was performed in the same conditions. A total of 200 snapshots, each representing a conformation, were collected. During the simulation, every 10 snapshots a conformation was ‘minimized’ and the potential energy of the resulting 20 complexes was calculated. The gain in potential energy (Δ*E*_n_) for each assembling starting structure type (N, H, B) was calculated with the formula Δ*E*_n_=*E*_n_ − (*E*_1_ × *n*)/*n* where *E*_n_ is the energy of the *n* assembled monomers, *E*_1_ is the energy of the monomer and *n* is number of monomers.

### Generation of contacts maps

Besides to final energies, MD simulations were also evaluated in terms of frequency of contacts (dissected into van Der Waals interaction and hydrogen bonds) of the residues in the pool of our amyloid models. An in house script was used to extract, from Amber MD results files, both distances and chemical relationships between atoms, allowing to collect the number of interactions thresholded at different distances during the time of simulations. The values reported in this work comprise interactions found at distances <3 Å, although shorter (stronger) interactions were also considered. This procedure allowed to create interaction maps indicating the most probable interaction sites. In particular, we calculated the contact maps referred to our model β-sheets (composed by 4 HEWL peptide, so encompassing 16×4=64 residues) when they interacted with themselves (forming the model amyloid core) or with polyphenols and ThT.

### Statistics

All mathematical and statistical processing as well as plotting and non-linear fitting was performed with QtiPlot [47]. ThT kinetic traces and associated errors represent the accumulation of at least three independent experiments. CR and CD spectra as well as AFM images are from one representative experiment.

## RESULTS

### HEWL and HEWL peptide aggregation kinetics

The aggregation kinetic of HEWL and HEWL peptide was monitored by ThT fluorescence assay, CR assay and AFM (Figure 1). The aggregation kinetic of the full length HEWL was previously described by Frare et al. [11], evidencing that HEWL undergoes X-Asp-based proteolysis when heated at acidic pH and results in different peptides responsible for the formation of amyloid-like fibrils. Figure 1(A) reports a typical aggregation kinetic in our conditions monitored with ThT: we evidenced a lag phase of 2–3 days during which the enzyme fragmented (as evidenced by SDS/PAGE, Supplementary Figure S1) and nucleated into aggregation seeds that started an elongation phase reaching a plateau in approximately 7–10 days. The typical HEWL peptide aggregation kinetics was described in Krebs and co-workers [35] as remarkably different (Figure 1E) with respect to that of full length HEWL, lacking the lag phase and resembling an exponential curve with a plateau stabilizing after 7–10 h. In both HEWL and HEWL peptide models the presence of cross-β structures typical of amyloid-like aggregation was confirmed by a typical red shift in CR assay (Figures 1B and 1F), by AFM experiments (Figures 1C and 1G) and, in HEWL peptide only, by CD (Supplementary Figure S2). HEWL fibrils collected after 300 h incubation (largely after the begin of the ThT plateau, to ensure reproducibility) ranged from 4 to 8.5 nm in diameter and from 0.25 to 3 μm in length, similar to those observed after 69 h incubation at pH 2.0 and 57°C by Arnaudov et al. [12]. HEWL peptide fibrils showed diameters ranging from 6.0 to 8.5 nm and lengths from 0.1 to 6.5 μm, similar to those previously reported in electronic microscopy studies [28]. All the results described above were reproducible when 1% or 5% v/v DMSO was added in the aggregation reaction, a condition met when aggregation was performed in the presence of polyphenols (results not shown).

### Polyphenol solubility and stability in experimental conditions

**Figure 2:**
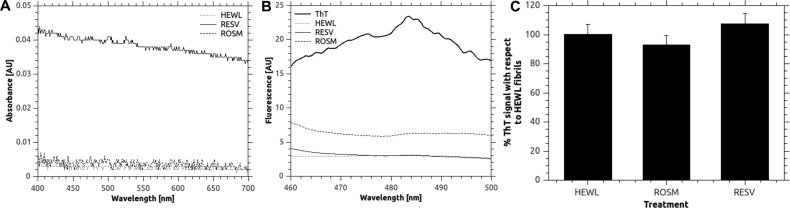
Rosmarinic acid and resveratrol do not disturb ThT fluorescence signals (**A**) Absorbance spectra of HEWL, rosmarinic acid (ROSM) and resveratrol (RESV) indicating that little or no scattering occurs due to limited solubility of the compounds. (B) Fluorescence spectra of the same compounds excited at 440 nm, indicating that no emission occurs at 485 nm, where ThT shows the amyloid-associated peak. (C) Fluorescence signals of ThT pre-incubated with preformed HEWL fibrils upon the addition of rosmarinic acid and resveratrol, indicating that no competition occurs between ThT and the tested compounds. In the latter, *y*-axis contains fluorescence values normalized by ThT signal. Error bars represent the S.D. of three independent measurements.

Since polyphenols are known to suffer from limited solubility, we firstly monitored this feature at 1 mM concentration, pH 2 and 65°C (the conditions we used during HEWL or HEWL peptide aggregation). A photometric measurement of the light scattering at 600 nm evidenced that both resveratrol and rosmarinic acid were soluble in our experimental conditions (Figure 2A). Mass spectrometry further confirmed the chemical stability of the molecules in the reaction conditions, evidencing that no fragmentation occurred during the aggregation process (results not shown).

### Effect of polyphenols on HEWL and HEWL peptide aggregation

HEWL incubation in presence of resveratrol and rosmarinic acid was followed by ThT assay and eventually checked by CR and AFM at late plateau time (300 and 30 h for HEWL and HEWL peptide respectively). ThT fluorescence evidenced that in the presence of rosmarinic acid the signals did not grew during aggregation time for either HEWL or HEWL peptide (Figures 1A and 1E). On the contrary, control samples and samples in the presence of resveratrol showed the typical aggregation kinetic (sigmoid for HEWL, exponential for HEWL peptide), terminating in a plateau phase (Figures 1A and 1E). Neither resveratrol nor rosmarinic acid proved to interfere with the fragmentation process required to prime aggregation of HEWL (not required for HEWL peptide), as evidenced by SDS/PAGE analysis (Supplementary Figure S2). Their inhibition values were calculated as the percent of ThT fluorescence signal with respect to untreated controls according the plateau value obtained from fitting (Figures 1A and 1E). As reported in Table 1, rosmarinic acid showed a marked reduction in both HEWL (83.5±3.6%) and HEWL peptide (85.6±10.1%), while in both cases resveratrol proved almost no reduction. The AFM analysis of rosmarinic acid-treated HEWL samples at plateau showed small particles with 1–3 nm diameter mixed with large flat aggregates with similar height (Figure 1D), completely different from the mature fibrils observable in aggregated HEWL controls (Figure 1C). Similarly, HEWL peptide showed small round particles of approximately 2–3 nm in diameter (Figure 1H), completely different from fibrils observed in aggregated HEWL peptide controls (Figure 1G). In order to ensure the stability of the rosmarinic acid inhibitory effect, HEWL peptide aggregating mixture was allowed to stabilize for 6 months at room temperature. AFM demonstrated that, although HEWL alone presented long, unbranched amyloid-like fibres, the presence of rosmarinic acid induced the formation of nearly spherical aggregates with diameters of approximately 25–30 nm (Supplementary Figure S3), compatible with the observation that a destabilization of the cross-β structure impair the elongation of fibrils, leading to a dramatic and stable reduction of the fibril burden. It is important to note that such small particles, as well as long-term stabilized HEWL fibrils, were non-toxic to SHSY-5Y neuroblastoma cell line (results not shown) [48].

**Table 1:** Summary of the effects of polyphenols on HEWL and HEWL 49–64 peptide (PEPT) aggregation Values for compounds are calculated as percentage reduction in ThT fluorescence signals (ex. 440 nm, em. 485 nm) with respect to plateau signals obtained by fitting of HEWL and HEWL peptide kinetic curves.

Compound	HEWL	PEPT
RESV	1.9±0.1	1.7±0.1
ROSM	83.5±3.6	85.6±10.1

### Polyphenols competition with ThT on preformed fibrils

Since polyphenols are optically active molecules, due to their aromatic rings, we evaluated possible optical artefacts in the working conditions used for ThT assay (Figure 2). We dissolved both rosmarinic acid and resveratrol at 10 μM concentration (the same used when in the presence of ThT for the aggregation assay) in PBS (ThT buffer) and acquired absorbance spectra in the range 400–700 nm as well as fluorescence spectra (slit size 5 nm) in the range 460–500 nm upon excitation at 440 nm (slit size 5 nm). Resveratrol did not absorb or scatter at the tested wavelength as compared with PBS alone or HEWL (Figure 2A). Rosmarinic acid gave a very low and broad signal, insufficient to generate optical artefacts in fluorescence measurements (Figure 2A). When excited at 440 nm, resveratrol showed no emission at the tested wavelengths with respect to both PBS alone and HEWL (Figure 2B). Rosmarinic acid gave rise to a slight increase in emission, although very reduced with respect to that observed for ThT (Figure 2B). These results evidenced that optical artefacts were not relevant in our measurement conditions.

It is now well described that polyphenolic compounds may compete with ThT or other amyloid probes for binding to aggregates [49,50]. We therefore tested this feature in our aggregation conditions. We found that the ThT signals on pre-formed HEWL fibrils were not significantly affected by rosmarinic acid addition (signal variation −8%, *t*-test *P*=0.1, *n*=9) nor by resveratrol (signal variation +7%, *t*-test *P*=0.09, *n*=9) (Figure 2C). These assays, together with the optical properties detailed above, implicitly supported the validity of using plateau values for estimating a percentage of inhibition and also confirms a substantial absence of competition of the tested molecules with the ThT dye for amyloid binding.

### Design of amyloid core scaffolds

In order to explain such different behaviour between rosmarinic acid and resveratrol we approached the problem using MD on a reconstructed amyloid core entirely composed by HEWL peptide monomers. To the best of our knowledge, no 3D structure is currently available for HEWL or HEWL fragments, apart from a very short region from human lysozyme [15]. CD analysis (Supplementary Figure S4) suggested that the peptide fully aggregates into assemblies composed by structures containing β-sheets, with no other residual structure. Accordingly, we started a semi-manual procedure to design different β-sheets by juxtaposing HEWL peptide monomers in a β-strand conformation.

**Figure 3:**
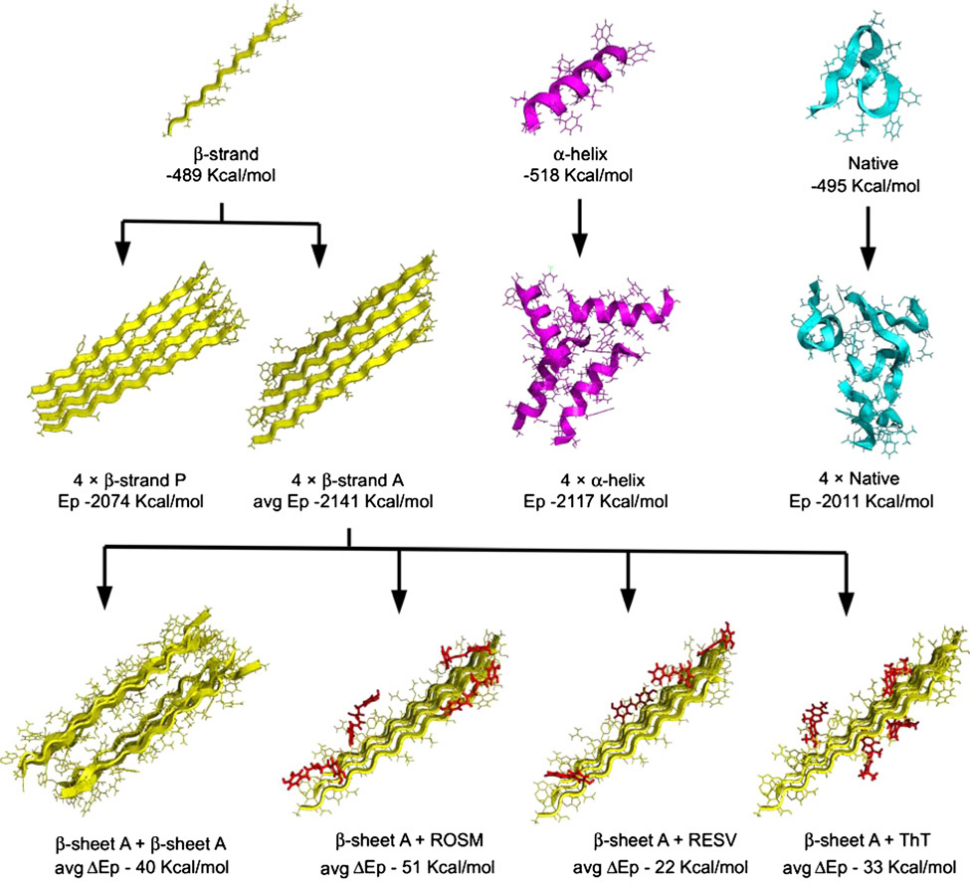
Scheme of the building of the amyloid core model and proposed MD-based inhibition assay strategy Top row: three monomeric conformations were initially built using DSV, namely β-strand, α-helix and native (obtained from HEWL taken from the PDB structure 2VB1 [35]). Middle row: the quaternary structure assembly was simulated by adding monomers one by one using AutoDock. α-Helix and native assemblies were used for energy references, verifying that all β-sheets conformations presenting alternating or in phase orientations of side chains were energetically favoured. Bottom row: two juxtaposed β-sheets gave rise to the amyloid core model. Its energy was compared with that obtained by the addition of polyphenols (ROSM, rosmarinic acid and RESV, resveratrol) or ThT to investigate their ability to compete with fibril formation.

We tested different number of participating peptides in several combinations of native (N, extracted from the original HEWL crystal structure), helix (H, the most stable conformation for a peptide *in silico*), or β-strand (B, involved in β-sheets and cross-β amyloid structures) conformations. When stacking B monomers, parallel (BP) or antiparallel (BA) directions of the strands were evaluated, also exploring different orientation of the side chains (namely BA1, with all side chains in the same orientation, BA2, with side chains with alternating orientations and BAa, with energy minimized combination of orientations obtained from AutoDock). For each tested structure we used molecular docking to find favourable initial poses, then we used MD in a minimization/heating/dynamic simulation protocol. Each result was evaluated in terms of total energy of the structure or energy per monomer (see Figure 3 for a schematic representation of the building process).

**Figure 4:**
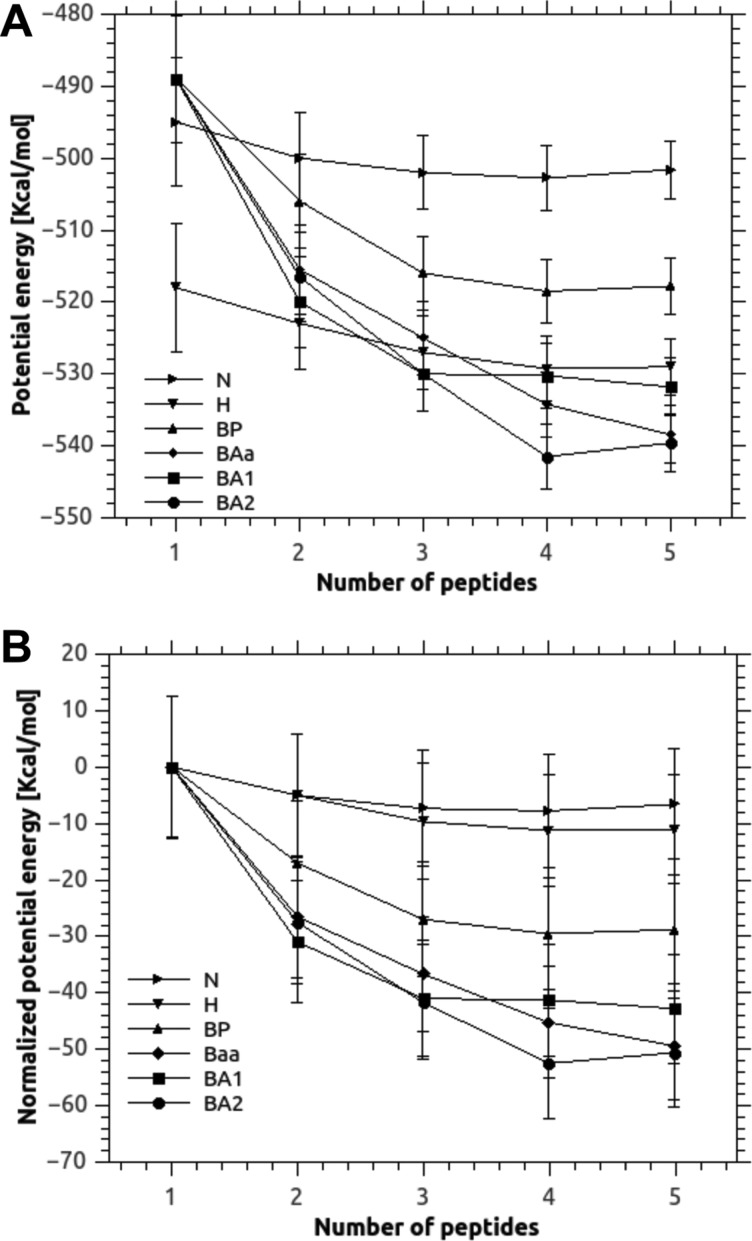
Initial building of the amyloid core model: potential free energies associated to various types of HEWL peptide assemblies Identical monomers in three different starting conformations, namely native (minimized with no constrains), α-helix or β-strand, were allowed to stack and interact to form an initial sheet. The accumulation of up to five monomers were followed my MD resulting in six different types of stacks: N (native stack), H (helix stack), BP (all-parallel β-stack), BAa (alternate antiparallel β-stack, minimized orientation of side chains), BA1 (alternate antiparallel β-stack, all side chains in the same direction) and BA2 (alternate antiparallel β-stack, all side chains in opposite directions). See the figure for a graphical visualization of the structures with four monomers, generally resulting as stabilized in terms of gain in energy. (**A**) Potential energy (Ep) of the assembling structures; (**B**) variation of potential energy (ΔEp) during the assembly.

Structures likely to give rise to sheets were selected according to two main criteria: (i) a constant relative gain in energy following addition of monomers and (ii) an overall energy lower than that achieved by a hypothetical assembly composed by monomers in α-helix conformation (by far the most stable initial conformation of our 16 residue long peptide). As shown in Figure 4(A), the gain in polymerization (normalized by the number of monomers) usually stabilized after the addition of a 4th peptide monomer. The most stable conformations were found to contain antiparallel β-strands. In particular, the conformation showing side chains at alternating faces (labelled as 4 × β-strand A in Figure 3), was found to be the most stable, with a relative energy gain of −2166±17.8 kcal/mol (−539.6 kcal/mol per monomer) although other BA conformations were found to be acceptable. The BP conformations were found to have energy, on average, 40 kcal/mol lower than that of the reference α-helix assembly, and therefore such assembly was considered as energetically disfavoured. This result was supported by the deconvolution of CD spectra of HEWL peptide after 46 h aggregation (Supplementary Figure S3) performed with the BestSel server [50], indicating a 57% of antiparallel β-sheets and almost no signal of parallel β-sheets (preliminary result). It is noticeable to observe that the ensemble built-up by strands in a conformation similar to that observed in the original, full-length HEWL, showed to be the less stable among the tested conformations.

A core fibril packaging was then built by imposing a face-to-face interaction between identical pairs of the three isomers of tetrameric β-sheets using MD to estimate the total energy. At this stage, the BP-based β-sheet was also evaluated to verify that this configuration was disfavoured also in the core fibril structure. Ten initial conformations for each pairing were semi-manually posed and then analysed by MD. We found that all antiparallel β-sheets paired with an energy 100 kcal/mol lower, on average, than parallel β-sheets (Figure 4B). Pairs of antiparallel conformations, although arising from different β-sheets, were found to stabilize in a number of conformations with relatively similar energy (within 1% each other), indicating that several types of fibrils, equally likely, could be formed. This led to a definitive pool of stable conformations, with energy gains with respect to separated β-sheets of −26±25, −65±25 and −28±25 kcal/mol for the BAa, BA1 and BA2 conformations, respectively, as reported in Table 2.

**Table 2:** Summary of the energies observed during the design of the amyloid core model Ep_β-sheet_: potential energy of a β-sheet composed by four strands. Ep_core_: potential energy of the cross-β fibril core composed by two β-sheets containing four strands each. ΔEp_core_: potential energy gain associated to β-sheet assembly into the amyloid core, calculated with the formula ΔEp_core_=Ep_core_ − 2 × Ep_β-sheet_. Labels are as follows: BP (all-parallel β-stack), BAa (alternate antiparallel β-stack, minimized orientation of side chains), BA1 (alternate antiparallel β-stack, side chains in the same direction) and BA2 (alternate antiparallel β-stack, side chains in opposite directions). All values are expressed as kcal/mol.

β-Sheet type	Ep_β-sheet_	Ep_core_	ΔEp_core_
BP	−2074±18	−4211±25	−63±22
BAa	−2137±18	−4300±35	−26±25
BA1	−2121±18	−4307±36	−65±25
BA2	−2166±18	−4359±35	−28±25
BA (avg)	−2141	−4322	−40

**Figure 5:**
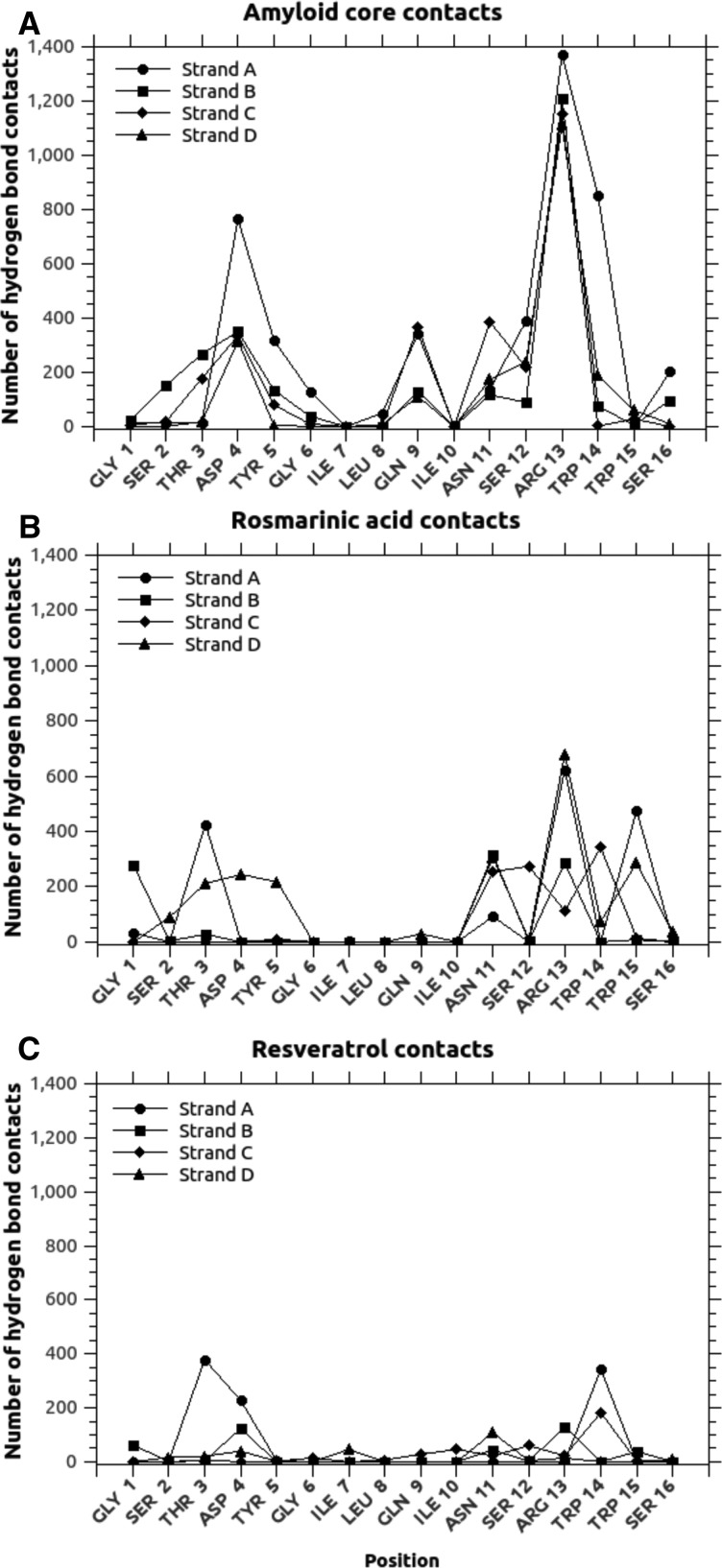
Hydrogen bond maps Hydrogen bond maps of our model 4-stranded β-sheet interacting with (**A**) a second, identical model β-sheet, (**B**) rosmarinic acid or (**C**) resveratrol. The positions of the interacting residues are reported in the *x*-axis (referred to a single monomeric 49–64 HEWL peptide) and the number of hydrogen bonds found during MD simulation by the interacting element (estimated at a distance within 3 Å) are reported on the *y*-axis. The four strands of the β-sheet are indicated by circles, squares, diamonds and triangles for strands A, B, C and D respectively.

**Figure 6:**
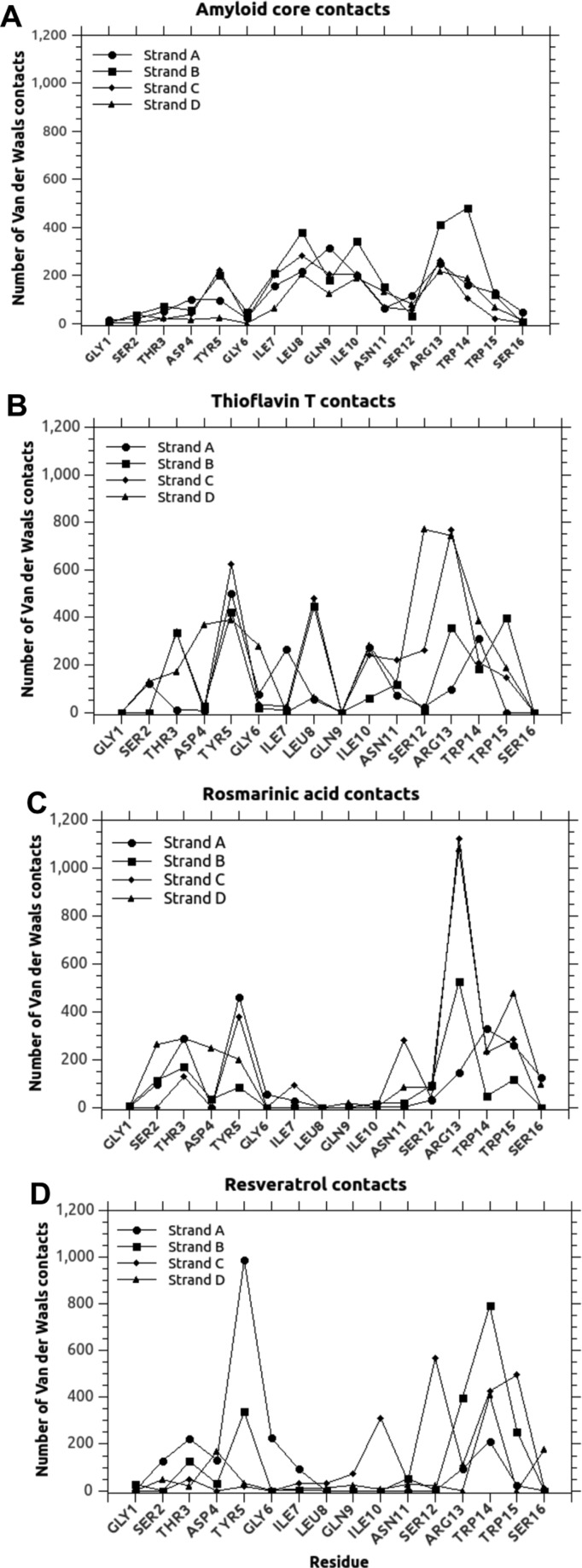
van der Waals interaction maps van der Waals interaction maps of our model 4-stranded β-sheet interacting with (**A**) a second, identical model β-sheet, (**B**) ThT, (C) rosmarinic acid or (**D**) resveratrol. The positions of the interacting residue are reported in the *x*-axis (referred to a single monomeric 49–64 HEWL peptide) and the number of van der Waals interactions found during MD simulation by the interacting element (estimated at a distance within 2.3 Å) are reported on the *y*-axis. The four strands of the β-sheet are indicated by circles, squares, diamonds and triangles for strands A, B, C and D respectively.

This pool was then used as a set of possible amyloid cores to be used as a testing ground for MD studies on resveratrol and rosmarinic acid as negative and positive effector in amyloid inhibition respectively. The path used to build the amyloid core model, as well as the energies resulting from MD, is depicted in Figure 3. From the analysis of hydrogen bond contact maps (Figure 5), obtained by counting the number of hydrogen bonds occurring between interacting side chains of paired sheets (pool) during the MD simulation, it emerged that the residue Arg^13^ of each strand is the major responsible of the stabilization of the model amyloid core, with minor contributions of Asp^4^ and Trp^14^ of the strand A, Gln^9^ of the strands B and C and Asn^11^ of the strand C. The van der Waals interaction maps (computed as for hydrogen bonds) further indicated a relevant contribution of the internal hydrophobic region (from Ile^7^ to Ile^10^) to the stabilization of the assembly (Figure 6). This core interaction spanning positions 7–10 was also confirmed by the consensus amyloid server prediction AmylPred2 [51], as shown in Supplementary Figure S5.

### MD of polyphenol interaction with growing β-sheets

AutoDock and MD (1 ns) were used to establish the binding energy and the most probable conformation(s) for the binding of polyphenols to peptide monomers in order to evaluate whether a competition could occur during the initial formation of the β-sheet.

The energy gain obtained by binding to rosmarinic acid or resveratrol was related to the concentration of polyphenols used. The stoichiometry ratios (peptide/polyphenol) used for the experimental measure was of 1:1. At stoichiometry ratios of 1:4 peptide/polyphenol the calculated energy gain was 12 and 19 kcal/mol for rosmarinic acid and resveratrol respectively. In both cases, energy gains were largely not sufficient to compete with the addition (stacking) of a new monomer (45–50 kcal/mol) required for the initial formation of the β-sheet (Figure 3). Such data suggested that a mechanism of inhibition based on an impairment of the formation of the β-sheet by blocking β-strand stacking should be excluded. In addition, the initial, most favourable, docking position from AutoDock indicated that both polyphenols did not form hydrogen bonds with atoms of the backbone, that primarily drive the association of protein secondary structure, but rather they interacted with side chains in orientations that did not obstacle the stacking of the strands.

### MD of polyphenol interaction with amyloid core

MD was then used to study the binding of polyphenols to the model amyloid core. Polyphenols were added for docking (at stoichiometry ratios of 1:1peptide/polyphenol) into preformed 4-strand β-sheets to determine favourable initial poses, and then a 1 ns MD simulation was run.

In both cases, as expected from previous results, AutoDock determined that the best binding positions involved interactions with specific side chains and not with backbone atoms (Figure 3, bottom row). MD indicated that the energy gain in having rosmarinic acid bound on a side of the β-sheet is more than double than that of having resveratrol. The average values obtained from the bound between the three types of preformed 4-strand β-sheets (described above), were −59 kcal/mol for rosmarinic acid and −26 kcal/mol for resveratrol. Considering that the average value obtained by the addition of two 4-strand β-sheet (as in the formation of the model amyloid core described above) involved an energy gain of −40 kcal/mol, it was reasonable to conclude that rosmarinic acid, but not resveratrol, was able to compete with the formation of the cross-β structure (Table 3). The analysis of hydrogen bond maps also confirmed this stronger interaction. In fact, rosmarinic acid was found to frequently form hydrogen bonds with the side chains of the β-sheets (5788 times for all models types during the simulation time, lower but comparable with the 6457 contacts of the sheet-to-sheet interaction in the amyloid core model detailed above), most noticeably with Thr^3^ of the A strand, Arg^13^ of the A and D strands and Trp^15^ of the A strand, plus a general preference for the 11–16 region (Figure 5B). No interaction was observed with backbone atoms. Resveratrol was also found not to bind backbone atoms. In addition, it showed only 2012 hydrogen bonds formed with the preformed β-sheet for all model types (during the 1 ns simulation), preferentially with Thr^3^ of the A strand Trp^14^ of A and D strands. Such positions were not involved in the stabilization of the amyloid core, thus reinforcing the observation that resveratrol is ineffective in inhibiting amyloid aggregation.

**Table 3:** Potential energy associated to amyloid inhibition Values represent the average gain in binding energy of test compounds to all the β-sheet of the model amyloid cores. For reference, the most favourable addition of second β-sheet (generating the full core), involved an average energy gain of −40 kcal/mol. All values are expressed as kcal/mol.

Compound	ΔEp_core_
RESV	−26±19
ROSM	−59±19
ThT	−33±19

Taken together, the results showed a clear agreement to what experimentally observed using all the amyloid probes and spectroscopic investigations, indicating that the MD approach we set up was effective in shedding lights into the molecular mechanism of amyloid inhibition by rosmarinic acid both in HEWL peptide or in the full length HEWL from which the extracted peptide was described as a major constituent of the amyloid core [11].

### Polyphenols does not compete with ThT for the amyloid core

Simulations similar to that used for polyphenols were also performed using ThT as a ligand. MD results indicated that ThT interacted with the β-sheet with an energy intermediate between rosmarinic acid and resveratrol (−33 kcal/mol, average value for three types of preformed 4-strand β-sheets described above), indicating a possible inhibitory effect, at least at the tested stoichiometry. Nevertheless, the interaction maps were found to be completely different with respect to those of rosmarinic acid. Of primary importance, ThT proved not to form hydrogen bonds with the amyloid core. When van der Waals interaction were monitored, ThT showed a broader spectrum of contacted residues and an overall higher number of interactions with respect to polyphenols (11454 total contacts for all model types compared with 8674 and 7260 for rosmarinic acid and resveratrol, respectively, in the whole 1 ns MD simulation), as shown in Figure 6. Such difference was found to be mostly related to contacts in the central hydrophobic region of the strands, almost completely neglected by polyphenols. This result was in agreement with the experimental evidence concerning the competition of polyphenols with ThT for amyloid binding (Figure 2). In fact, the addition of rosmarinic acid on preformed HEWL amyloid fibrils previously bound with ThT was observed to have a very limited competition effect. Such competition, that is currently regarded as a major confounding evidence in amyloid research [49,50], has for long time being neglected, seriously compromising the interpretation of inhibition effects.

## DISCUSSION

In the current study we managed to explain, using MD, the different behaviour of two polyphenols, rosmarinic acid and resveratrol, on amyloid aggregation of HEWL upon heat-induced acid fragmentation. We demonstrated that the two molecules display extremely different effects on amyloid aggregation, the former being highly inhibitory, the latter completely inactive. Since we wanted to study such properties using computational techniques we had to face two different problems: (i) the absence of a reference structure to use as a model and (ii) the need of a model that is simple enough in terms of participating atoms, to be fully evaluated in MD. Both problems were overridden by the use of the well described amyloidogenic 49–64 HEWL peptide (16 AA long), after a preliminary experimental verification that all the results obtained with full length HEWL applied to this simplified model.

To the best of our knowledge, no 3D structure of amyloid aggregates of HEWL is available or suitable to our needs. HEWL was found to form spherical aggregates (spherulites) large in diameter and composed by an isotropic assembly of 1–2 μM fibrils. Such structures were observed in several other amyloidogenic proteins [52] and are hard to study by morphological techniques such as TEM or AFM. The work of Yagi et al. [53] using microbeam X-ray diffraction on fibrils from full-length HEWL suggested conformations that did not encompass the well described [11] amyloidogenic 49–64 region we used in MD and biophysical experiments. Little hints were also attainable by the work of Zou et al. [54], describing that HEWL aggregates may exist in both parallel and antiparallel β-sheet conformation leading or not to productive fibril assembly, depending on the experimental conditions.

Since we did not have a reference structure of HEWL amyloid, we chose to use a supervised approach to build a model amyloid core *ab initio*. To this aim, we followed three fairly naïve assumptions: (i) the internal structure of amyloid is a β-sheet; (ii) cross-β interactions are at the basis of amyloid; (iii) the aggregation follows a path in which monomers build up sheets and then sheets assembled into fibrils. Such simplified assumptions were found to give energetically robust results, evidencing at least three important considerations.

First, we found that the formation of the β-sheet was favoured until a 4th strand stacked, the addition of a 5th sheet not giving a supporting gain of energy. This is consistent with the current knowledge on protein structures, that were not generally observed with extended sheets, apart from functional or structural specificities such as in silk fibroin [55] due to unfavourable dihedral angles that impose a twist in the sheet and therefore a limitation in planarity of the resulting structure. This result is also supported by the aforementioned work by Zou et al. [54], describing that HEWL aggregates formed at lower temperatures are populated by off-pathway oligomers with antiparallel conformations. We may speculate that our model (that we termed model amyloid core) is representative of an initial assembly that is not sufficiently stable to elongate into fibrils and whose formation can be effectively hampered by the addition of exogenous compounds.

Secondly, we found that the face-to-face packing of a second sheet further stabilized the overall structure, favouring the formation of the cross-β structure typical of amyloid. In this set-up, interdigitated side chains builds up a zipper that confers planarity to both strands, thus favouring the formation of the assembly.

Thirdly, we found that β-sheets formed by antiparallel strands were relatively more stable than those obtained from parallel strands. This is not in contrast with the vast majority of literature on disease-related proteins, reporting that amyloid is built-up by parallel, frequently in register β-sheets [56]. In fact, peptides containing a single β-strand more frequently forms fibrils using an antiparallel conformation, supporting the idea that short peptides extracted from the main chain are more frequently found in antiparallel conformation, whereas full peptides containing two strands are more stable in a parallel conformation [56]. Examples of fibrils with antiparallel configurations are largely documented, such as the NFGAIL fragment of IAPP [57], the D23N IOWA mutant of Aβ1–40 [58], or series of small aggregating fragments from the Aβ peptide containing amyloid strands [59–62]. On the other hand, the amyloid core of HEWL, among others, was recently investigated for the construction of novel nanomaterials [15]. The human sequence IFQINS (corresponding to ILQINS in HEWL), described as the most aggregation prone, was allowed to self-assemble into fibrils ad then crystals were resolved by X-ray crystallography, revealing the stacking of zipped β-sheets with a parallel orientation of the β-strands. The authors claimed that such structure was largely the most stable. From our simulation studies, an antiparallel orientation of the β-strands resulted the most probable, and the more likely to form fibrils from the HEWL peptide 49–64. Although dissonant, the two results are not to be considered in disagreement for at least two reasons: (i) the length of the IFQINS peptide (6 AA) that can form a highly stable hydrophobic zipper structure with no interference by surrounding residues and (ii) the absence of charged residues that do not introduce electrostatic effects in the overall structure. The consensus amyloid stretch predictor AmylPred assigned to a short region spanning positions 7–10 a high propensity to form the typical amyloid zipper, but the parallel conformation of the sheets would be disfavoured by the residues Asp^4^ and Arg^12^ flanking such region. On the contrary, an antiparallel orientation favoured both the hydrophobic interactions in the zipper and the electrostatic interactions between polar and charged residues. Indeed, it has been demonstrated that the nature and conformation of amyloid structures are largely variable and highly depend on the sequence length, composition and other peculiar features, as reviewed by Sawaya et al. [63].

As evaluated by MD, we found that the association energies between ligands and elements of the model amyloid β-core could easily explain their overall effect on aggregation. The energies of 1:1 binding of a single β-strand with itself or with resveratrol and rosmarinic acid was clearly in favour of the β-sheet growth. This held true also when the ratio of strand:polyphenol was tested at 1:4, meaning that the formation of β-sheets could not be blocked by the addition neither of rosmarinic acid nor of resveratrol. What could rosmarinic acid (not resveratrol) do was indeed to compete with the pairing of two sheets. In a monomer:polyphenol 1:1 stoichiometry, the energy associated to rosmarinic acid interaction was comparable to that of a second sheet, whereas that of resveratrol was found to be much lower. Accordingly, a true competition might occur only with rosmarinic acid, that is what experimental data suggested. Using the energy gain of β-sheets self-assembly in model amyloid core as a cut-off and the overall hydrogen bond mapping, we could rationalize that molecules that do not bind to single β-sheets with an energy comparable to that of self-assembly or bind at positions not involved in the same stabilizations of the paired sheets, could be considered as unlikely to give inhibitory effects.

Although our results suggest our method as a potential prediction tool (e.g. by ranking the testing compounds by a combination of binding energy and position), we tend not to encourage such perspective but rather to suggest to use the model as a rule of thumb for calling as inhibitory a molecule that have an energy higher or at least comparable to that of the model amyloid core, provided that the contact maps, in terms of hydrogen bonds, is comparable. Nevertheless, examples have been documented of molecules that, upon binding, have the unpredictable (and undesirable) behaviour of favouring aggregation due to an ordering effect. As an example, a recent work on Aβ (1–40) peptide reports that ThT can be a promoter of aggregation, rather than an inhibitor as its well-known binding capability may have suggested [64].

Since we found that the energy associated to β-sheet stacking is hard to be competed, due to the network of hydrogen bond interactions between backbone atoms of the strands, the inhibitory effect should, in general, be searched in the hampering of the β-sheet pairing through strong interactions with side chains. This can unequivocally lead to the conclusion that, as the sequences of amyloid cores varies widely among the different amyloid system known so far, although rationalized by several observations and algorithms [65,66], there must be an extreme specificity in finding good inhibitors for different proteins.

Our future work will be aimed at generating amyloid core models for different amyloid related proteins and testing our system in additional situations with respect to those performed in this pilot work. In particular, we will use, where available, the proposed crystals of fibrils as the starting ground for the building of the computational framework. When not available, we will use the wealth of computational methods developed to identify amyloidogenic regions in protein sequences to do a *de novo* building and further verify whether this approach is feasible in other different and less described contexts.

## CONCLUSION

In this work we have developed a strategy for the study and the characterization of small molecule interaction with amyloid-related proteins. Although several structural assumptions were made (taking into account literature and protein structure theories), our results were fully supported by experimental data. Our computational framework can describe the effect of a small ligand on HEWL peptide from two different perspectives, both related to amyloidogenicity, i.e. (i) the competition with β-sheet elongation or (ii) the competition with β-sheet association. Both activities play a pivotal role in amyloid development and may give rise to different effects. Our results supported our general strategy of considering as equally likely structures derived from a pool of conformations rather than just the most stable one, and computing binding energies as the result of an association between such pooled constituents. The strategy introduced in this work represents an initial step towards the ability of rationalizing the effects of drugs aimed at inhibiting and preventing amyloid deposits, giving new insights into the development of new, rationally designed, anti-amyloid drugs.

## Abbreviations

CR: Congo red
HEWL: hen egg white lysozyme
MD: molecular dynamics
ThT: Thioflavin-T
